# Grassy Silica Nanoribbons and Strong Blue Luminescence

**DOI:** 10.1038/srep34231

**Published:** 2016-09-26

**Authors:** Shengping Wang, Shuang Xie, Guowei Huang, Hongxuan Guo, Yujin Cho, Jun Chen, Daisuke Fujita, Mingsheng Xu

**Affiliations:** 1 College of Information Science & Electronic Engineering, State Key Laboratory of Silicon Materials, Department of Polymer Science and Engineering, Zhejiang University,Hangzhou 310027, P. R. China; 2 State Key Laboratory of Silicon Materials, School of Materials Science and Engineering, Zhejiang University, Hangzhou 310027, P. R. China; 3 Nano Characterization Unit, National Institute for Materials Science,1-2-1 Sengen, Tsukuba 305-0047, Japan; 4 Nano Electronics Materials Unit, WPI Center for Materials Nanoarchitectonics (MANA), National Institute for Materials Science (NIMS), 1-1 Namiki, Tsukuba, Ibaraki 305-0044, Japan

## Abstract

Silicon dioxide (SiO_2_) is one of the key materials in many modern technological applications such as in metal oxide semiconductor transistors, photovoltaic solar cells, pollution removal, and biomedicine. We report the accidental discovery of free-standing grassy silica nanoribbons directly grown on SiO_2_/Si platform which is commonly used for field-effect transistors fabrication without other precursor. We investigate the formation mechanism of this novel silica nanostructure that has not been previously documented. The silica nanoribbons are flexible and can be manipulated by electron-beam. The silica nanoribbons exhibit strong blue emission at about 467 nm, together with UV and red emissions as investigated by cathodoluminescence technique. The origins of the luminescence are attributed to various defects in the silica nanoribbons; and the intensity change of the blue emission and green emission at about 550 nm is discussed in the frame of the defect density. Our study may lead to rational design of the new silica-based materials for a wide range of applications.

Silicon dioxide (SiO_2_) is one of the key materials in many modern technological applications. SiO_2_ is the gate dielectric layer that forms the basis of field-effect transistors on the present-day Si integrated circuits^1^, and current metal oxide semiconductor (MOS) transistors use SiO_2_ films of about 1.3 nm in thickness as the gate dielectric. SiO_2_ is also widely explored as an antireflection coating for photovoltaic solar cells^2^. It has been recently demonstrated that a silica layer on top of a silicon absorber significantly reduced the temperature of the underlying absorber under sunlight due to radiative cooling^3^. In addition, silica-based materials can be worked as catalysts^4^,^5^,^6^ for pollution removal and for biomedical applications^7^.

Owing to the fundamental scientific and technological significance of silica, there is great interest in development of novel structures of silica and understanding the basic structure-property relationships in silica-based materials^8^,^9^,^10^,^11^,^12^,^13^,^14^,^15^. It has been found that ultrathin layers of silica on metals occur either as ordered hexagonal structures or as disordered arrangements of non-hexagonal rings. In particular, the crystalline phase of two-dimensional (2D) silica consists of two registered layers of SiO_4_ tetrahedra; and the amorphous 2D silica resembles the 2D continuous random network^8^,^14^,^15^. These 2D silica layers can be synthesized in a chemical vapor deposition (CVD) furnace or grown by molecular beam epitaxy on various transition metals, such as Mo, Ru, Pt, Ni, Pd, and Cu^8^,^15^. Were the 2D silica layers isolated from metal substrates, like graphene^16^ and other 2D layered materials^17^, their intrinsic properties and enormous applicability could be investigated at large^12^.

Here, we report the accidental discovery of free-standing grassy silica nanoribbons directly grown on SiO_2_/Si platform which is commonly used for field-effect transistors fabrication, without providing other precursor. The formation of silica nanoribbons is catalyzed by the co-existence of chromium (Cr) and sulfur (S) in a CVD system. We have performed rigorous analyses of the structure, chemical composition, and cathodoluminescence (CL) properties of the silica nanoribbons. The amorphous silica nanoribbons exhibit strong blue luminescence at about 467 nm. Despite yet unknown technologically relevant performances of the silica nanoribbons as well as 2D silica structures, the understanding of their basic physicochemical properties and controllable growth mechanism may lead to rational design of the new silica-based materials for a wide range of applications.

## Results

**Figure 1:**
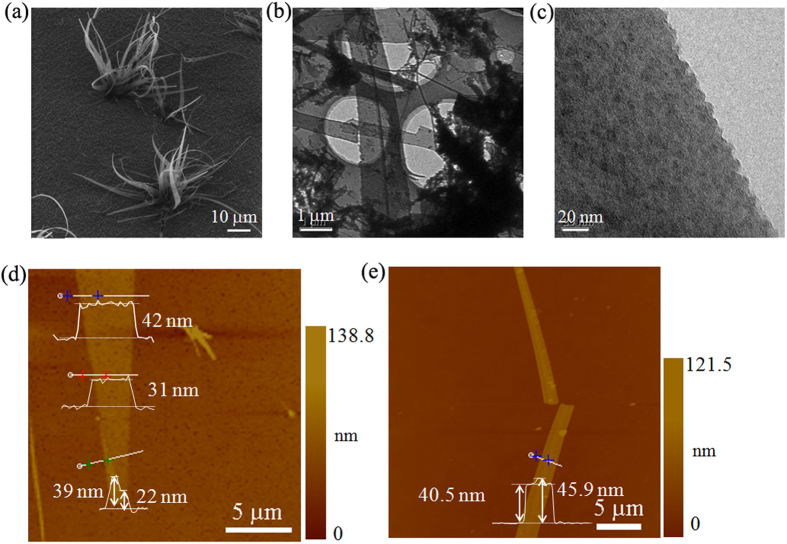
Morphology of silica nanoribbons. (**a**) Typical SHIM image of bunches of silica nanoribbons. (**b**,**c**) TEM images of silica nanoribbons. (**d**,**e**) Topographic AFM images and line-profiles showing the thickness of nanoribbons.

Figure 1a shows a representative Helium ion microscopy (HIM)^18^ image of the silica nanoribbons grown on SiO_2_/Si substrate. The gatherings of nanoribbons with a length of tens of micrometers and varying widths resemble bunches of *chlorophytum comosum* planting into the ground. We further transferred several nanoribbons from the SiO_2_/Si substrate onto transmission electron microscope (TEM) Cu meshes to investigate the fine microstructures of the nanoribbons. As displayed in Fig. 1b, the silica nanoribbons are electron-transparent. No ordered structure is observed in the high-resolution TEM image (Fig. 1c and Figure S1 in Supp. Info.), suggesting an amorphous nature of the silica nanoribbons. We had also annealed the nanoribbons for 2 h at 1000. However, amorphous to crystalline structure transition did not occur. The amorphous feature of the nanoribbons is confirmed by the X-ray diffraction (XRD) pattern of the samples (see Figure S2, Supp. Info.). This phenomenon could be attributed to the multilayered structure of the silica nanoribbons, since it has been reported that growing additional layers on top of the crystalline silica monolayer would finally result in amorphous silica films^19^. The thickness of individual nanoribbons based on atomic force microscopy (AFM) measurements ranges from 5.4 nm (Fig. 1e) to approximately 50 nm, becoming thinner along the growth direction of the grassy nanoribbons (Fig. 1d,e).

**Figure 2:**
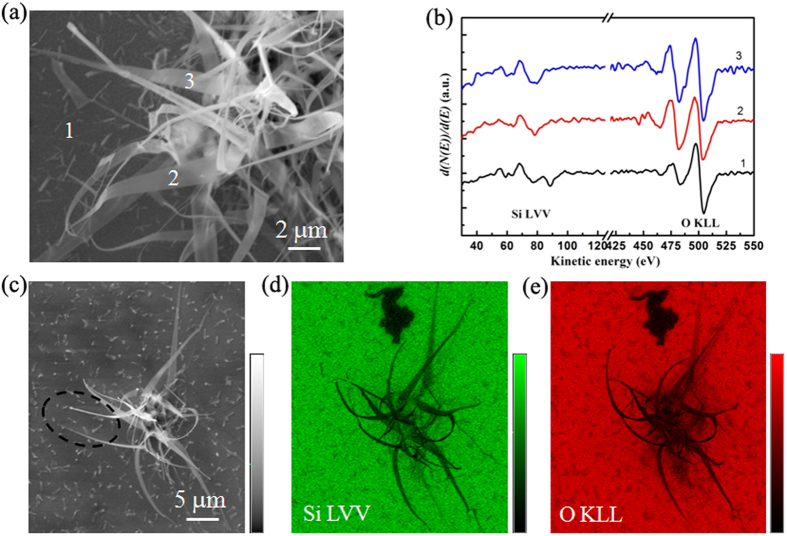
AES characterization of silica nanoribbons. (**a**) Scanning secondary electron image of the sample, showing the locations of AES spectra acquiring area. (**b**) Differential AES spectra acquired at positions “1”, “2” and “3” marked in (**a**). (**c**) Scanning secondary electron image of the sample, showing the locations of AES mapping. (**d**) Si LVV Auger electron map acquired in (**c**). (**e**) O KLL Auger electron map acquired in (**c**). The oval in (**c**) marks movement of the nanoribbon under electron-beam as compared to the positions observed in (**d**,**e**).

We characterized the chemical composition and Si oxidation states of the silica nanoribbons by employing local scanning Auger electron spectroscopy (AES) technique^16^,^20^,^21^,^22^. The Si LVV AES peak position and shape are extremely sensitive to the Si oxidation state^21^. The main peak for elemental Si is centered around 90 eV while that for SiO_2_ is located at 76 eV^21^,^23^. Figure 2b depicts the AES spectra acquired at different sample locations in Fig. 2a. At the substrate region, i.e., #1 position, the Auger electron signals of both the elemental Si and the Si oxidation states for SiO_2_ were detected. By contrast, only the latter was detected on the nanoribbons, i.e., #2 and #3 positions. Figure 2c,e show the Auger electron maps of the Si LVV (Fig. 2d) and O KLL (Fig. 2e), elucidating the chemical distribution of the elements in the silica nanoribbons. Besides, the X-ray photoelectron spectroscopy (XPS) results corroborate the formation of silica nanoribbons (Figure S3, Supp. Info.). Raman spectrum (Figure S4, Supp. Info.) and Fourier transform infrared spectroscopy (FTIR) (Figure S5, Supp. Info.) techniques were also used to analysized the silica nanoribbons in ambient air.

## Discussion

**Figure 3:**
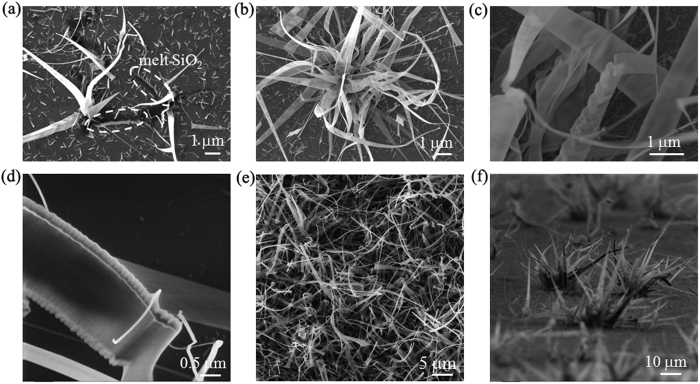
Formation of silica nanoribbons and various fine structures. (**a**) SEM image showing initial formation of silica nanoribbons at the melted crack-wall sites. (**b**) SEM image showing a bunch of silica nanoribbons together with nanowires. (**c**) SEM image showing nanoribbons with different morphologies and deformation of nanoribbons by electron-beam. (**d**) SHIM image highlighting different edge structures of nanoribbons. (**e**) SHIM images showing dense nanoribbons. (**d**) tilt-view of bunched silica nanoribbons.

We found the formation of silica nanoribbons when heating very thin Cr films deposited on SiO_2_/Si substrates under S atmosphere. However, without either Cr or S, no silica nanoribbon could be obtained. It is a big surprise, to some degree, because a thin Cr layer is normally an adhesive layer of the source/drain electrodes for field-effect transistors on SiO_2_/Si platform^24^. In the literature, the production of monolayer or bilayer 2D silica depended only on the metals and no S was present in their reaction systems^11^,^12^,^13^,^14^,^25^,^26^,^27^,^28^. Therefore, we believed that Cr and S acted as the dual co-catalysts for the growth of silica nanoribbons. Furthermore, on the basis of the morphological observation of the different phases of the growth, the growth mechanism of silica nanoribbons is most likely to be as follows (see Fig. 3 and Figure S6 in Suppl. Info.). Cracks were firstly generated in the SiO_2_ layer on Si with the assistance of Cr and S at high temperature (Fig. 3a); melting of the SiO_2_ occurred at the wall of the SiO_2_ cracks; and nanoribbons grew *in-situ* at the crack sites. It is also sound from the observations that the Si and O sources for the formation of silica nanoribbons were originated from the melted SiO_2_. The formed silica nanoribbons exhibit various features. Some nanoribbons like a braid (Fig. 3b,c), and some have wedge-shaped edges (Fig. 3d). It is also observed that long silica nanowires with size of about 20 nm (Figure S6e, Supp. Info.) were produced together with the nanoribbons (see Figs 2c–e and 3). In addition, short silica rods were also produced, lying on the substrate (see Figs 2c–e and 3). The generation of these silica nanostructures on the SiO_2_ substrate is rather interesting and the grassy appearance of the silica nanostructures is quite astonishing. The silica nanoribbons are bendable. Moreover, the nanoribbons are sensitive to the exposure of electron beam. As demonstrated in the sequentially acquired AES mappings in Fig. 2c–e, the nanoribbons highlighted in the oval were movable under the electron beam irradiation, which indicates the possibility to manipulate the nanoribbons by electron beam. Also, excessive exposure of electron beam could damage the nanoribbons as observed in Fig. 3b,c. Our grassy silica nanoribbons show distinct morphological characteristics from previous reported silica nanostructures such as nanowires^29^ and twisted nanobelts and nanosprings^30^ synthesized by a thermal evaporation method at 1300 °C, porous nanostructures synthesized by complicated solution chemical reactions^31^, and silica nanotubes synthesized by a template-directed method^32^.

**Figure 4:**
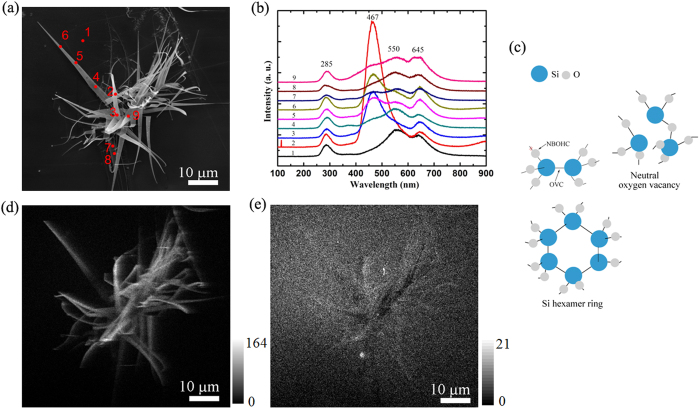
CL characteristics of silica nanoribbons. (**a**) SEM image showing the positions where the CL spectra were acquired in (**b**) and emission maps in (**e**,**f**). (**b**) CL spectra acquired at different positions. (**c**) Schematical illustration of an OVC and a NBOHC, neutral oxygen vacancy, and Si hexamer ring. (**d**) Blue band map corresponding to (**a**). (**e**) Green band map corresponding to (**a**).

Defects in SiO_2_ exert significant influences on its properties such as dielectric performance and luminescence. The defect structure of SiO_2_ is extremely sensitive to ionizing radiation^33^,^34^. Several kinds of defects in amorphous SiO_2_ are optically active and can be studied by luminescence spectroscopy. We investigated the defect-induced luminescence properties of the grassy silica nanoribbons by CL spectroscopy, which is a frequently used technique for high spatial resolution and high-sensitivity detection of defect centers in materials. Figure 4 displays the CL characterization results. All the CL spectra (#2–#9 in Fig. 4a,b) acquired from the different sample locations of nanoribbons possess luminescence features at around 285 nm (4.35 eV, UV band), 467 nm (2.60 eV, blue band), 550 nm (2.25 eV, green band), and 645 nm (1.92 eV, red band)^33^. No blue emission can be detected from the substrate (#1). These luminescent bands are common in silica and the specific luminescence centers related to them have been well documented^35^,^36^,^37^,^38^, originating from local atomic rearrangement that deviates from the SiO_4_ tetrahedra expected for a perfect silica matrix. The red emission band ascribed to the nonbridging oxygen hole centers (NBOHCs)^39^,^40^ (see Fig. 4c). The UV emission band can be originated from particular kinds of oxygen vacancy centers (OVCs) such as the discoordinated Si or neutral oxygen vacancy (Fig. 4c)^35^,^37^.

The most striking change in the present set of CL spectra is the luminescent intensity of the blue and green emissions (Fig. 4a,b). At the substrate location (#1), no blue emission appears, in line with previous study on unirradiated amorphous SiO_2_^41^. However, at the apex (#2 as well as #3) of the upright, free-standing silica nanoribbon (Figs 3e and 4a), very strong blue emission is observed. This comparison suggests that the blue emission is most likely to be orginated from surface features of the high surface area silica nanoribbons rather than the bulk features of silica. Thus, on the basis of previous study on the blue emission of silica nanostructures^42^,^43^,^44^,^45^, the observed blue emission here could be associated with a defect pair consisting of dioxasilirane (=Si (O2)) and a silylene (=Si:). As for the green emission, this may be associated with oxygen-related defects such as silanone (=Si=O) or dioxasilyrane^40^.

The intensity change of the blue and green emissions is clearly illustrated by the luminescent spectra acquired at the #4, #5 and #6 locations. These phenomena can be explained based on the origins of the luminescence as discussed above. On the one hand, we confirmed the remarkable defective nature of the silica (SiO_x_) nanoribbons by TEM-based energy dispersive spectroscopy (EDS) measurements. An atomic ratio ranging from 1.54 to 2.09 between O and Si, typically smaller than 2.0, was obtained for the SiO_x_ nanoribbons (Figure S7, Supp. Info.). This substoichiometric characteristic suggests the existence of oxygen vacancies in the silica nanoribbons, leading to the strong blue emission. On the other hand, as shown in the AES spectra (Fig. 2b), elemental Si signal was detected at the substrate, which indicates the existence of Si aggregates at the substrate surface resulted from the silica production as addressed above. Therefore, strong green luminescence also appears at the substrate. The intensity mappings of the blue and green emissions are displayed in Fig. 4d,e, respectively. The map of blue emission matches well with the silica nanoribbons (Fig. 4a). The reason resulting in the intensity change of blue and green bands might be the variation of the oxygen content in the silica nanoribbons. Although a quantitative correlation between the oxygen content and the emission intensity is difficult to be concluded at this stage, the lower oxygen content, responsible to the blue emission, could suggest a higher component of silicon aggregates, responsible to the green emission, in the silica nanoribbons. In fact, the OVCs could be described as Si-Si links in the form of dimers, timers or hexamers^42^,^46^. In this perspective, the very weak blue emission at #4 is likely due to the higher content of oxygen than those at #5 and #6 locations.

**Figure 5:**
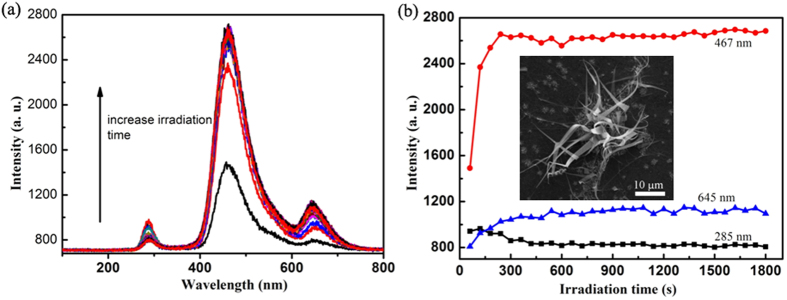
Electron irradiation effect on CL spectrum of silicon nanoribbons. (**a**) Evolution of CL spectrum with electron irradiation time. (**b**) Intensity change of UV band, blue band, and red band.

We investigated the CL properties of a gathering of silica nanoribbons as a function of irradiation time (Fig. 5). The CL emission was excited by continuous irradiation over the observation field with an electron beam of energy 5.0 keV and current 0.1 nA. The very weak green emission of the first collected CL spectrum might be due to the relatively small substrate area because irradiated area is almost fully covered by the silica nanoribbons and silicon aggregates responsible for the emission is not as sensitive to the irradiation as the OVCs. The intensity of the red emission increased with the irradiation time from 0 s to about 600 s; and the UV emission first increased and then decreased within the first 300 s. In the case of the blue emission, the intensity escalated rapidly to a constant intensity within the first 240 s. With irradiation, green emission was being overlapped by the strong blue emission. The trend of the irradiation effect on the luminescence of the nanoribbons is similar to the previous observations on amorphous SiO_2_ films^41^. Nevertheless, the previous amorphous SiO_2_ films^41^ did not possess the green emission as detected on our substrate, and we also failed to observe the blue emission from the substrate. The different behaviours of the UV and blue emissions upon the electron irradiation indicate different origins of the emissions. These results indicate that the electron irradiation significantly increases the number of luminescent centers of the silica nanoribbons, strongly dependent on their stoichiometries or absolute deficiency of oxygen atoms over silicon atoms^47^,^48^.

In summary, we produced silica nanoribbons by a facile method. The silica nanoribbons look like *chlorophytum comosum*. The silica nanoribbons have a strongly characteristic blue luminescence at about 476 nm as compared to the SiO_2_ substrate. Moreover, the silica nanoribbons are flexible and can be manipulated by electron-beam. Whatever the defects responsible for the emissions, this new structure of silica enriches our knowledge of silica-based materials. While the allotropes of bulk silica have made a great impact in fundamental and applied research, the exceptional properties revealed here herald that the grassy silica nanoribbons may present potential applications for, such as composite materials and solar cells as antireflection coating and radiative cooling.

## Methods

### Silica Nanoribbon Synthesis

We used chemical vapor deposition method to synthesize the silica nanoribbons. SiO_2_/Si substrate coated with a thin layer of Cr (99.95%) was used for silica nanoribbon growth under a sulfur atmosphere. Vapor of sulfur was produced by heating surfur powder. The growth temperature was about 820 °C. We tried to get crystalline silica by annealing the as-prepared silica nanoribbons at 1000 °C for 2 h under Ar atmosphere.

### Materials characterization

Characterizations of the silica nanoribbons were carried out by using an atomic force microscope (AFM, Digital Instrument Nanoscope IIIA), a powder X-ray diffractometer (XRD, Rigaku D/Max Ultima IV), a field emission scanning electron microscope (FESEM, Hitachi S-4800), a transmission electron microscope (TEM, FEI TECNAIG2 F20-TWIN) equipped with an energy dispersive X-ray spectroscope (EDS), and X-ray photoelectron spectroscopy (ESCALAB 250Xi system). The topographic images of the silica nanoribbons were acquired in the tapping mode. The SEM images were obtained on a Hitachi S4800 field-emission SEM system with an accelerating voltage of 3.0 kV. The XRD was operated at 40 kV, 40 mA for Cu Kα radiation (λ = 1.5418 Å). XPS was performed using Al Kα as the source and the C 1s peak at 284.8 eV as an internal standard. An acceleration voltage of 200 kV was used for EDS measurement. Raman spectrum was obtained by using Renishaw inVia system with a 532 nm laser in ambinet air. Fourier transform infrared spectroscopy (FTIR) was recorded on a Bruker Vector 22 spectrofluorometer in ambient air.

### Cathodoluminescence (CL) Characterization

CL measurement was performed by using field emission scanning electron microscope (FESEM;HITACHI SU6600) equipped with CL system (HORIBA MP32). An acceleration voltage of 5.0 kV and beam current of 0.1 nA were used for CL measurement.

### Scanning Auger Electron Spectroscopy

The AES measurements were performed at room temperature with a scanning Auger electron spectroscope (ULVAC-PHI model SAM650) with a cylindrical mirror analyzer. The takeoff angle of the instrument was 42°. AES spectra were acquired with a primary electron beam of 10 keV. The incident electron beam current for the AES spectra was about 2.0 nA, as calibrated with a Faraday cup before and after each measurement. Area-analysis mode can be chosen to acquire electron spectra. Acquiring of each elemental map (512 × 512 pixels) took ~3.5 h.

## Additional Information

**How to cite this article**: Wang, S. *et al.* Grassy Silica Nanoribbons and Strong Blue Luminescence. *Sci. Rep.* **6**, 34231; doi: 10.1038/srep34231 (2016).

## Supplementary Material

Supplementary Information
