# Supplementary material for: Grassy Silica Nanoribbons and Strong Blue Luminescence
Source: Sci Rep. 2016 Sep 26;6:34231. doi: 10.1038/srep34231 (PMC5035931; doi:10.1038/srep34231)
Supplement: Supplementary Information [file srep34231-s1.doc]

**Supplementary Information**

**Grassy Silica Nanoribbons and Luminescence Properties**

Shengping Wang^1,ζ^, Shuang Xie^2,ζ^, Guowei Huang^1^, Hongxuan Guo^3^, Yujin Cho^4^, Jun Chen^4^, Daisuke Fujita^3^ & Mingsheng Xu^1^,*

^1^ School of Information Science & Electronic Engineering, State Key Laboratory of Silicon Materials,Department of Polymer Science and Engineering, Zhejiang University,Hangzhou 310027, P. R. China

^2^ State Key Laboratory of Silicon Materials,School of Materials Science and Engineering, Zhejiang University, Hangzhou 310027, P. R. China

^3^ Nano Characterization Unit, National Institute for Materials Science,1-2-1 Sengen, Tsukuba 305-0047, Japan.

^4^ Nano Electronics Materials Unit, WPI Center for Materials Nanoarchitectonics (MANA), National Institute for Materials Science (NIMS), 1-1 Namiki, Tsukuba, Ibaraki 305-0044, Japan

^ζ^ S. Wang and S. Xie contributed equally to this work.

* msxu@zju.edu.cn


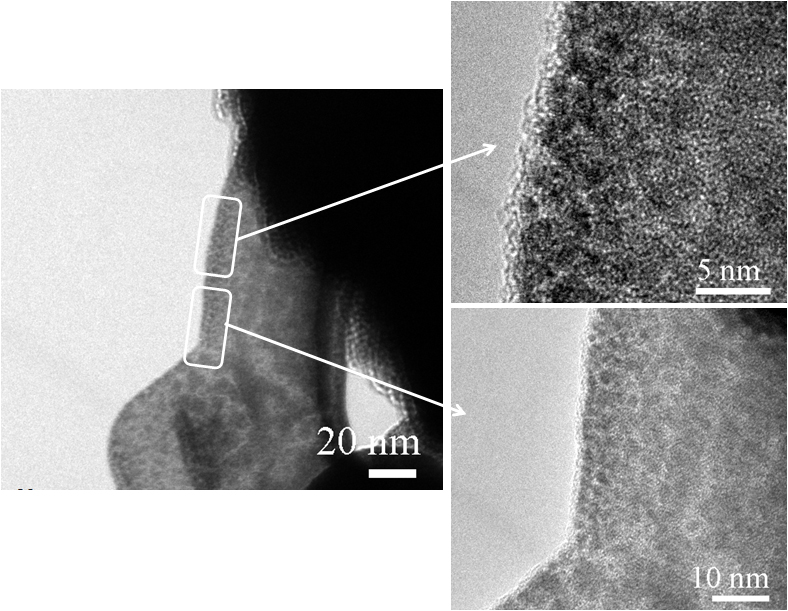


**Figure S1.** High-resolution TEM images of the edge of silica nanoribbons.


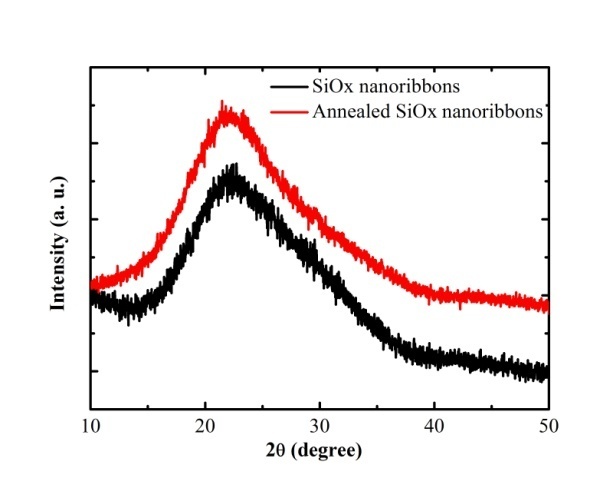


**Figure S2.** XRD patterns for as-prepared silica nanoribbons and nanoribbons after annealed for 2 h at 1000 °C. The results suggest amorphous silica nanoribbons.


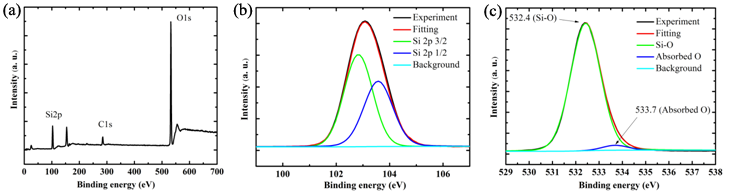


**Figure S3.** XPS patterns for silica (SiOx) nanoribbons. (a) Survey spectrum. (b) Si 2p spectrum. (c) O1s spectrum.

The XPS survey spectrum manifests that the composites contain Si and O elements. The Si 2p XPS spectra of the SiO_X_ nanoribbons are deconvoluted into two peaks representing the Si 2p 3/2 and Si 2p 1/2 levels. The energy difference between the Si 2p 3/2 and Si 2p 1/2 levels is ~0.6 eV as previous report ^1^. The O 1s spectrum of the silica nanoribbon is asymmetric, indicating that there are different chemical states of oxygen. The spectrum can be fitted with two peaks for oxygen atoms in different chemical states: oxygen in siloxane bonds (Si–O), oxygen from chemisorbed oxygen and perhaps some adsorbed water^2,3^. The results suggest formation of Si-O bond.


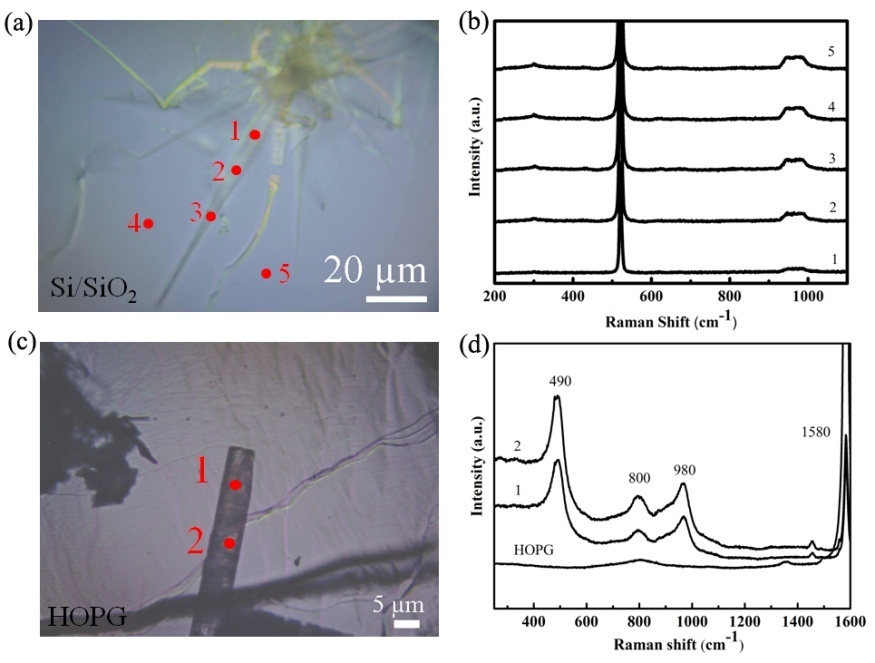


**Figure S4**. Raman characterization of silica nanoribbons. (a) Characterization of the silica nanoribbons on the SiO_2_/Si substrate used for the silica nanoribbons synthesis. (b) Raman spectra acquired at the position marked in (a). The Raman feature acquired at the silica nanoribbon is identical to that aquired at the substrate. (c) Characterization of the nanoribbon transferred onto HOPG substrate. (d) Raman spectra aquired at the the position marked in (c). The Raman peaks of the silica nanoribbon at 490 cm^-1^, 800 cm^-1^, and 980 cm^-1^ is the breathing mode of 4-membered rings (D_1_ line), the SiO_2_ network optical mode, and the vibration of the (OH)-group with respect to Si, respectively^4^. It is not clear why the silica nanoribbon on the grown SiO_2_/Si does not exhibit Raman signal of the silica nanoribbon at the present, which requires further investigation. Note that the measurement was performed in ambient air.


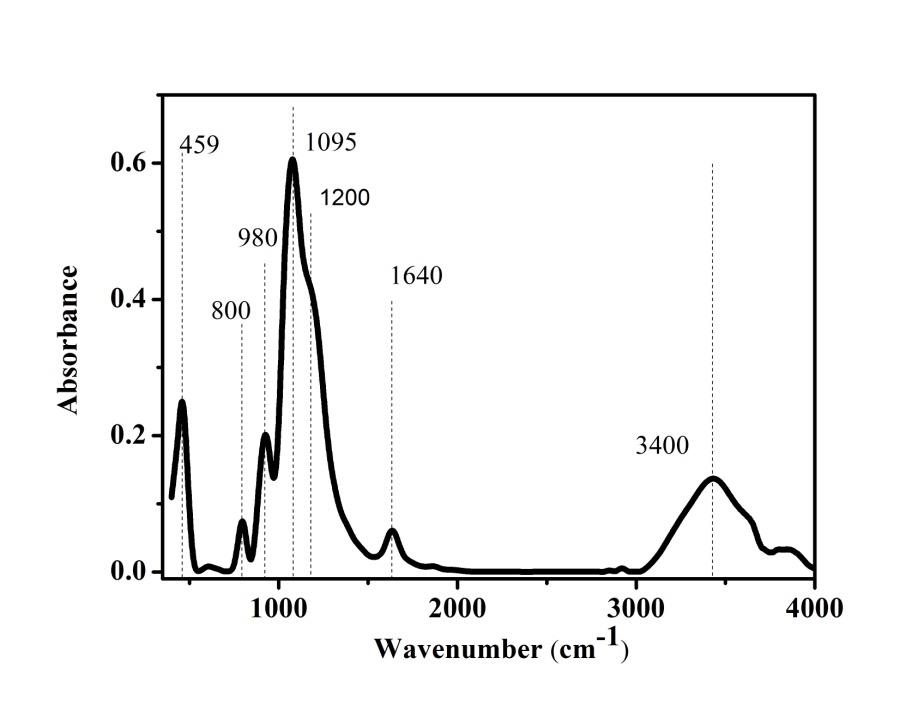


**Figure S5**. Fourier transform infrared spectroscopy (FTIR) characterization of silica nanoribbons. Several wide bands are observed^4^. The 459 cm^-1^ is the bending mode; 800 cm^-1^ is the symmetric stretching mode; 1095 cm^-1^ and 1200 cm^-1^ are the transversal and longitudinal asymmetric stretching, respectively. The 980 cm^-1^ is the (OH)–Si stretching mode. The broad band around 3400 cm^-1^ originates from the overlay of different O-H strecthing modes (adsorbed water, isolated, terminal and geminal silanols)^5^. Note that the measurement was performed in ambient air.


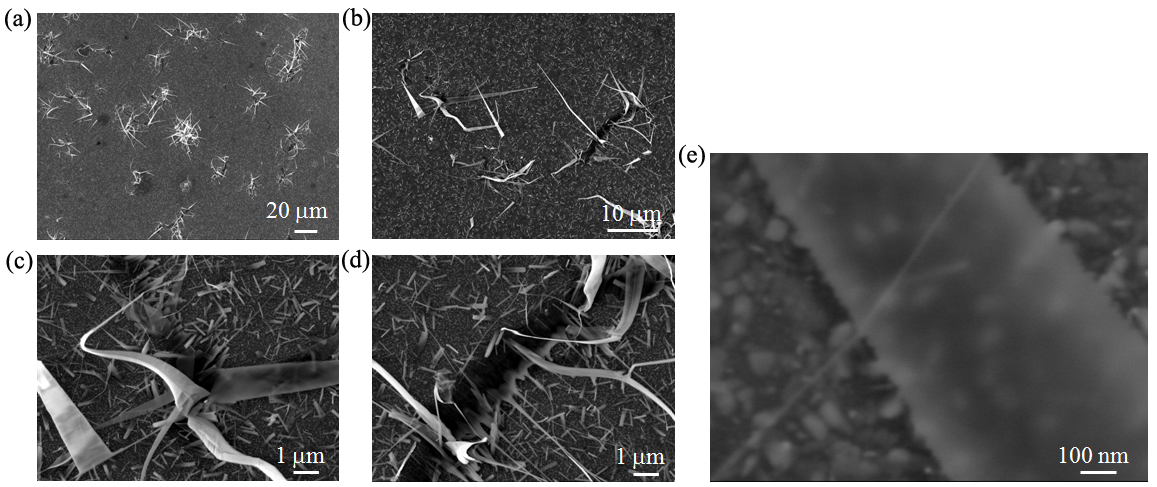


**Figure S6.** SEM images showing the different growth stages of silica nanoribbons, together with nanowires.


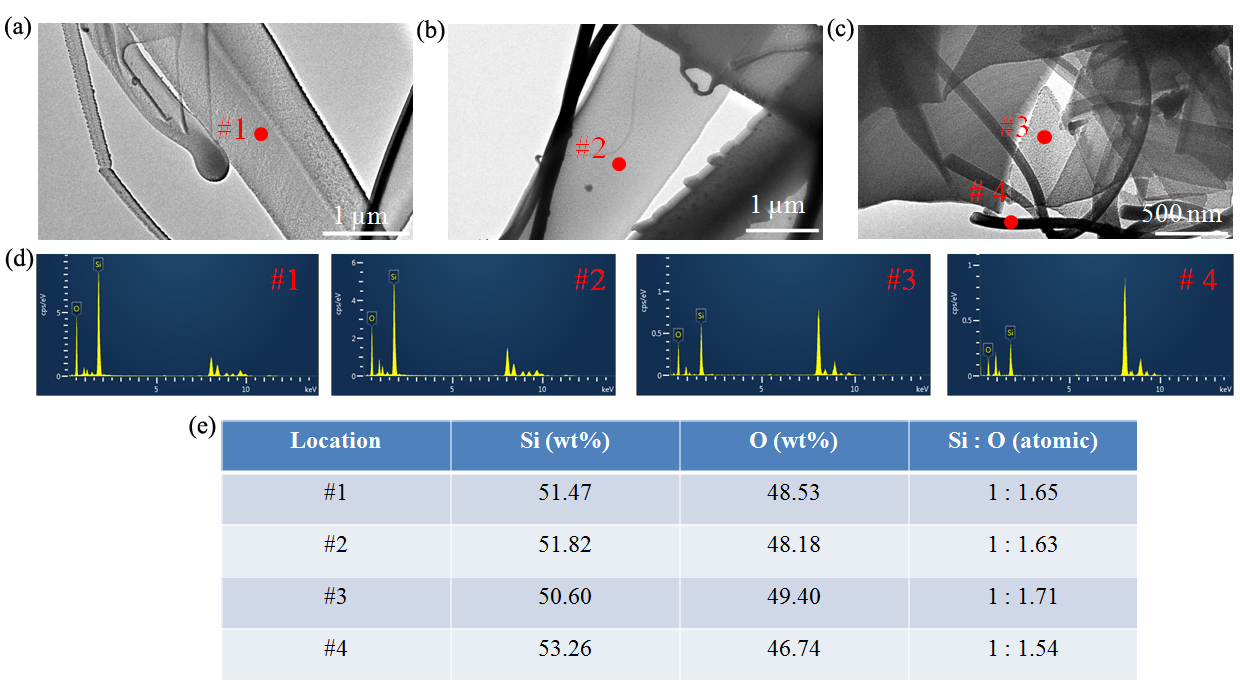


**Figure S7.** TEM and EDS characterization of silica nanoribbons. It is found that the ratio of silicon and oxygen atoms is different at different locations.

**References**

1. Pleul, D., Frenzel, R., Eschner, M. & Simon, F. X-ray photoelectron spectroscopy for detection of the different Si–O bonding states of silicon. *Anal. Bioanal.Chem.* **375**, 1276-1281 (2003).
2. Black, L., Garbeva, K., Stemmermanna, P., Hallamb, K. R. & Allenb, G. C. Characterisation of crystalline C-S-H phases by X-ray photoelectron spectroscopy. *Cem. Concr. Res.* **33**, 899–911 (2003).
3. Yueb, Z. R., Jianga, W., Wang, L., Gardner, S. D. & Pittman, C. U. Surface characterization of electrochemically oxidized carbon fibers. *Carbon* **37**, 1785–1796 (1999).
4. Spallino, L., Vaccaro, L., Sciortino, L., Agnello, S., Buscarino, G., Cannas, M. & Gelardi, F. M. Visible-ultraviolet vibronic emission of silica nanoparticles. *Phys. Chem. Chem. Phys.* **16**, 22028-22034 (2014).
5. Innocenzi, P. Infrared spectroscopy of sol–gel derived silica-based films: a spectra-microstructure overview. *J. Non-Cryst. Solids* **316**, 309-319 (2003).
